# Resident Endoscopy Experience Correlates Poorly with Performance on a Virtual Reality Simulator

**DOI:** 10.1055/s-0042-1743517

**Published:** 2022-03-03

**Authors:** Kurun Partap S. Oberoi, Michael T. Scott, Jacob Schwartzman, Jasmine Mahajan, Nell Maloney Patel, Melissa M. Alvarez-Downing, Aziz M. Merchant, Anastasia Kunac

**Affiliations:** 1 Division of General Surgery, Department of Surgery, Rutgers New Jersey Medical School, Newark, New Jersey; 2 Division of General Surgery, Department of Surgery, Rutgers Robert Wood Johnson Medical School, New Brunswick, New Jersey; 3 Division of Trauma and Surgical Critical Care, Department of Surgery, Rutgers New Jersey Medical School, Newark, New Jersey

**Keywords:** GI Mentor, colonoscopy, surgical, simulation, endoscopy, residency training

## Abstract

**Background** Endoscopy training has become increasingly emphasized during general surgery residency as reflected by introduction of the Fundamentals of Endoscopic Surgery (FES) examination, which includes testing of skills on virtual reality (VR) simulators. Although studies exist to assess the ability of the simulator to differentiate between novices and experienced endoscopists, it is not well understood how simulators can differentiate skills among resident cohort.

**Objective** To assess the utility of the VR simulator, we evaluated the correlation between resident endoscopy experience and performance on two VR simulator colonoscopy modules on the GI-BRONCH Mentor (Simbionix Ltd, Airport City, Israel).

**Methods** Postgraduate years 2 to 5 residents completed “easy” and “difficult” VR colonoscopies, and performance metrics were recorded from October 2017 to February 2018 at Rutgers' two general surgery residency programs. Resident endoscopy experience was obtained through Accreditation Council for Graduate Medical Education case logs. Correlations between resident endoscopy experience and VR colonoscopy performance metrics were assessed using Spearman's rho (ρ) correlation statistic and bivariate logistic regression.

**Results** Fifty-five residents out of 65 (84.6%) eligible participants completed the study. There were limited correlations found between resident endoscopy experience and FES performance metrics and no correlations were found between resident endoscopy experience and binary metrics of colonoscopy—ability to complete colonoscopy, ability to retroflex, and withdrawal time of less than 6 minutes.

**Conclusion** The VR simulator may have a limited ability to discriminate between experience levels among resident cohort. Future studies are needed to further understand how well the VR simulator metrics correlate with resident endoscopy experience.

Over the past several years, there has been an increased emphasis placed on the importance of endoscopy during general surgery training. This has been reflected by the Accreditation Council for Graduate Medical Education (ACGME) increasing the number of endoscopy cases required for general surgery trainees as well as the introduction of the American Board of Surgery (ABS) Flexible Endoscopy Curriculum, which culminates in the Fundamentals of Endoscopic Surgery (FES) examination. The ACGME and the ABS utilize resident operative experience as a proxy for procedural competence. As of 2018, the above-mentioned requirements must be met to sit for the general surgery boards. 1

The FES examination consists of a multiple-choice cognitive examination and hands-on skills test. 2 The latter is administered on a virtual reality (VR) endoscopy simulator—GI-BRONCH Mentor (Simbionix Ltd, Airport City, Israel). 2 The ABS notes that a simulator is not necessary to prepare for the FES skills test and that preparation can be achieved using resources already available at an institution. 1 However, recent studies have shown that clinical endoscopy experience alone may not be sufficient preparation for trainees to pass this examination. 3 4 In this vein, several institutions developed their own proficiency-based simulation curricula to train for the skills component of the FES examination. 5 6 7 These curricula have utilized physical 5 7 and/or VR simulators 6 for training.

As the FES skills examination is administered on the GI Mentor, there is a theoretical advantage of using this platform to prepare for this examination; it could be used to allow a resident to gauge their readiness to take the examination and practice their skills. One downside is that the specific skills modules tested on the FES examination are not publicly available. 8 There are other modules that can potentially be used to assess the same critical skills that the Society of American Gastrointestinal and Endoscopic Surgeons expects one to master during their training. Previous studies have aimed to study the ability of simulator metrics to differentiate endoscopists with varying clinical experience using some of these modules and found limited significant differences between novices and experts. 9 It is not yet well understood; however, how performance on the simulator can distinguish among resident cohort, and if current recommendations of endoscopy case numbers performed before taking the FES examination are adequate to achieve procedural proficiency.

To assess the utility of the VR platform, we aimed to evaluate the correlation between resident clinical endoscopy experience and performance on two selected VR colonoscopy modules at two general surgery residency programs. We hypothesized that performance on a simulated colonoscopy would be a poor way to discriminate between experience levels among resident cohort.

## Methods

### Setting and Participants

The resident endoscopy curriculum includes review of the FES online didactic curriculum during the postgraduate years (PGYs) 1 through 5, which was historically complimented by dedicated clinical endoscopy experience from PGY 2 through PGY 5. When the GI Mentor became available on the two Rutgers campuses, New Jersey Medical School (NJMS) and Robert Wood Johnson (RWJ), it was incorporated into the formal simulation curriculum at each general surgery residency training program. After this implementation, these data for this study were collected. Study participation was voluntary, and trainees were offered the same orientation to the simulator regardless of participation. The study took place from October 2017 through February 2018. PGY 2 through PGY 5 categorical residents at NJMS and RWJ general surgery residency programs were eligible for participation. The only exclusion criteria was performing deliberate practice on the simulator prior to study enrollment out of concern that the participants might thoroughly know the simulations, which would be an inaccurate reflection of endoscopic skill. Deliberate practice was identified as those residents who had either already taken the FES examination or used the same modules—the practice modules and/or case modules—to prepare for the examination.

### Interventions

Participants were asked to complete a survey to provide demographic information, rate their comfort level on a scale of 1 ( *not comfortable* ) to 5 ( *very comfortable* ) with performing upper and lower endoscopies, and remark on their prior experience with endoscopy simulators. Upper endoscopy experience was asked to participants as there are data that show a possible correlation between upper and lower endoscopy performances. 10 Participants were instructed to complete two standard cases provided by the GI Mentor (EndoBubble Case 1 and EndoBasket Case 1) to become familiar with the virtual interface.

Participants were tasked with completing the “easy” ( *First Module for Lower Gastrointestinal Endoscopy: Case No. 1* ) and “difficult” ( *First Module for Lower Gastrointestinal Endoscopy: Case No. 3* ) virtual colonoscopies. They: (1) took a picture of the ileocecal valve, (2) performed a retroflexion maneuver at the end of the procedure and took a picture for the “easy” VR colonoscopy, and (3) did not intubate the terminal ileum. Proctors gave participants 15 minutes to complete each colonoscopy. This 15-minute time limit was chosen to allow for ample time for even novices to complete both cases while considering the time constraints unique to general surgery. Proctors were general surgery residents in a research year and were well oriented to the details of the selected study cases.

### Outcomes Measured

Both VR colonoscopies tested four critical manual skills as outlined by the FES curriculum—navigation, loop reduction, retroflexion, and mucosal evaluation. The VR simulator automatically records metrics after the completion of every case. Metrics assessed for the “easy” VR colonoscopy included percentage of time the virtual patient was in pain (%), percentage of mucosal surface examined (%), time to reach the cecum (seconds), and total time (seconds). The amount of additional time that was spent performing the retroflexion maneuver was included by the simulator in the “total time” metric. Therefore, the metrics dependent on “total time”—calculated withdrawal time and efficiency—would not be accurate and were excluded. Each proctor recorded whether the colonoscopy was completed. Metrics assessed for the “difficult” VR colonoscopy included those of the “easy” VR colonoscopy along with efficiency of screening and withdrawal time. Withdrawal time (seconds) was calculated using the recorded time to cecum and total time metrics. For other binary metrics, the ability to successfully retroflex was assessed for the “easy” VR colonoscopy and having a withdrawal time > 6 minutes—which is commonly cited as a quality indicator for colonoscopy 11 —was assessed for the “difficult” VR colonoscopy.

Participants had their deidentified ACGME case logs downloaded from the secure ACGME Case Log System by the residency program coordinators at each site. All upper and lower endoscopy cases performed up until the date of study participation were counted. Upper endoscopy cases included all diagnostic and/or therapeutic endoscopies of the esophagus, stomach, and/or small bowel. Lower endoscopy cases included all diagnostic and/or therapeutic sigmoidoscopies or colonoscopies.

### Analysis of the Outcomes

Descriptive statistics for all variables are reported as counts with percentages for categorical variables and as medians with interquartile ranges for continuous variables. Correlations between clinical endoscopy experience—upper, lower, and total endoscopy cases—and continuous performance metrics were assessed using Spearman's rho (ρ) correlation statistic. The correlations between clinical endoscopy experience and binary performance metrics—the ability to complete a given colonoscopy, retroflex in the “easy” VR colonoscopy, and have a withdrawal time > 6 minutes in the “difficult” VR colonoscopy—were assessed using bivariate logistic regression. 11 A *p* -value of < 0.05 was considered significant.

SAS software Version 9.4 (Copyright 2018, SAS Institute Inc., Cary, NC) was used for statistical analysis.

### Institutional Review Board Statement

Approval from the Institutional Review Board was obtained from both Rutgers sites—RWJ and NJMS.

## Results

### Participant Characteristics

Fifty-five out of 65 possible residents (84.6%) met criteria and completed the study ( Table 1 ). While 20/55 (36%) of residents used a physical endoscopy simulator prior to participating in this study, only 4/55 (7%) had used a VR simulator. The number of total colonoscopies previously performed by the participants shows 24/55 (44%) falling within the lower end of 0 to 9 colonoscopies performed versus 4/55 (7%) in the upper end of 80 to 89 colonoscopies ( Table 2 ).

**Table 1:** Demographic data of participants

Demographic characteristic	Number	Percentage
Total number of participants	55	–
Institution		
NJMS	32	58%
RWJ	23	42%
Gender		
Male	35	64%
Female	20	36%
Age (y)		
25–29	18	33%
30–34	31	56%
35–39	4	7%
≥40	2	4%
Level of clinical training		
PGY 2	13	24%
PGY 3	14	25%
PGY 4	14	25%
PGY 5	6	11%
Laboratory resident (completed PGY 2)	5	9%
Laboratory resident (completed PGY 3)	3	5%
Dominant hand		
Right-handed	51	93%
Left-handed	4	7%

Abbreviations: NJMS, New Jersey Medical School; PGY, postgraduate year; RWJ *,* Robert Wood Johnson.

**Table 2:** Participant survey results

Participant survey question		Number	Percentage (%)
Physical simulator use	Never used	35	64
	Used	20	36
VR simulator use	Never used	51	93
	Used	4	7
Upper endoscopy comfort level a	1	11	20
	2	9	17
	3	23	43
	4	9	17
	5	2	4
Lower endoscopy comfort level a	1	17	31
	2	13	24
	3	19	35
	4	5	9
	5	0	0
Total endoscopies performed	0–9	24	44
	10–19	8	15
	20–29	7	13
	30–39	5	9
	40–49	4	7
	50–59	1	2
	60–69	0	0
	70–79	2	4
	80–89	4	7

Abbreviation: VR, virtual reality.

a Responses in these sections were 54 rather than 55 due to one participant not completing this part of the survey.

### The Effect of Resident Endoscopy Experience on the Performance of Virtual Colonoscopy

For the “easy” VR colonoscopy, the only significant correlation found was between number of upper endoscopies performed and percentage of mucosa evaluated ( Table 3 ). However, there was no correlation between resident performed endoscopies—upper, lower, and total—and the other measured metrics for performance on the “easy” VR colonoscopy. For the “difficult” VR colonoscopy, the significant correlations found were between the number of upper, lower, and total endoscopies performed and two metrics—efficiency of screening and time to cecum. With the exception of the above, there were no other significant correlations found between resident performed endoscopies—upper, lower, and total—and measured metrics for performance on the “difficult” VR colonoscopy. Most importantly, resident endoscopy experience had no correlation with the ability to complete the colonoscopy, the ability to successfully retroflex, or with withdrawal time for the “easy” and “difficult” VR colonoscopies. Previous VR simulator use was associated with a slightly higher percentage of mucosa evaluated in the “easy” case (90.50 [88.50–91.50] vs. 85.00 [82.00–88.00]; *p* = 0.04). Prior physical simulator use (329.00 [226.00–475.00] vs. 212.00 [130.00–321.00]; *p* = 0.03) and right handedness were correlated with a longer withdrawal time.

**Table 3:** Correlations of resident endoscopy experience with performance metrics of VR colonoscopy

	Easy virtual colonoscopy			Difficult virtual colonoscopy		
	Resident endoscopy experience			Resident endoscopy experience		
	Upper	Lower	Total	Upper	Lower	Total
Continuous metrics						
% time of patient in pain	0.22	0.07	0.13	0.11	−0.13	−0.02
Time to cecum	−0.13	−0.20	−0.16	0.37 a	−0.29 a	−0.38 a
Total time	0.10	−0.14	−0.01	−0.22	−0.15	−0.22
% of mucosa evaluated	0.30 a	0.14	0.27	−0.0002	0.11	0.09
Withdrawal time				0.30 a	0.18	0.26
Efficiency of screening				0.33 a	0.31 a	0.39 a
Binary metrics						
Ability to complete colonoscopy	1.001 (0.929–1.078)	1.022 (0.973–1.075)	1.010 (0.978–1.043)	0.983 (0.903–1.069)	0.979 (0.936–1.023)	0.987 (0.957–1.018)
Ability to successfully retroflex	0.998 (0.926–1.077)	1.017 (0.968–1.069)	1.008 (0.976–1.041)			
Withdrawal time > 6 min				1.056 (0.992–1.124)	1.033 (0.997–1.069)	1.023 (0.999–1.049)

Abbreviation: VR, virtual reality.

Notes: Continuous metric correlations reported as Spearman's rho (ρ) correlation coefficient. Binary metric correlations reported as odds ratios (95% confidence interval).

a *p* -Value < 0.05.

## Discussion

As one of the largest studies that looked at the correlation of documented resident endoscopy experience with performance on a simulator, our study showed limited correlations between upper, lower, and total endoscopy experience and metrics. Overall, we did not find any correlation with endoscopy experience and the main metrics—completing the colonoscopy, the ability to successfully retroflex, withdrawal time—and most of the FES examination skills. We found that for the “easy” VR colonoscopy, there was a correlation between one's upper endoscopy experience and percentage of mucosa evaluated; several studies have shown a correlation between total endoscopy numbers and this particular metric, 12 13 but there do not appear to be any reports of an isolated correlation between upper endoscopy experience and percentage of mucosa evaluated. Oddly enough, one study reported that those with more experience saw less mucosa. 9

Given the correlation between endoscopy experience—upper, lower, and total—and faster time to cecum with the more “difficult” VR colonoscopy and the fact that the critical difference between the two cases was that the “difficult” one was prone to loop formation, this suggests that those with more clinical endoscopy experience are better able to recognize loop formation and/or reduce loops, thus resulting in a faster time to cecum. The metric of efficiency is a combination of time to cecum and percentage of mucosa evaluated; since there was no correlation with endoscopy experience and percentage of mucosa evaluated, this suggests that the correlation between endoscopy experience and efficiency is heavily influenced by the correlation between endoscopy experience and time to cecum. Prior physical model use was correlated with a longer withdrawal time. The reason for this may be an increased familiarity with the expectation to inspect the mucosa carefully for lesions during withdrawal. Although right handedness was correlated with a longer withdrawal time, this finding should be interpreted with caution as there were 51 right-handed versus only 4 left-handed residents.

Analysis of our dataset reveals that the number of endoscopy cases completed by our residents showed a right skew, with the majority falling into the 0 to 9 cases category. Part of the reason for this was the reduced number of PGY 5. However, this may be a more realistic distribution of trainee experience. Studies using the GI Mentor test modules showed an ability to distinguish between novices and experienced endoscopists. 14 15 16 However, the number of procedures used to distinguish between these groups often far exceeded the minimum case numbers required by the ACGME for a graduating general surgery trainee (e.g., 200, 500, and/or 1,000 procedures). There are several lines of evidence that suggest the required case minimums are not sufficient to achieve the requisite skills to pass the FES examination. Other studies found that the minimum number of total cases associated with a passing score on the manual skills portion of the examination was 103. 3 The American Society for Gastrointestinal Endoscopy (ASGE) suggests much higher thresholds than the ACGME and that at minimum 275 colonoscopies and 130 upper endoscopies should be performed prior to assessment for competency. 17 One possible reason for why we found a limited correlation between case numbers and VR colonoscopy metrics despite our trainees being on track to meet the ACGME case minimum by graduation is that the case numbers recommended by the ACGME are not adequate to achieve procedural competency. One way to test this hypothesis would have been to have several faculty experts use the simulator and compare their metrics with those of the residents who were tested.

There were limitations in our study. For one, our study primarily focuses on a limited number of components of validity evidence. According to the most current edition of the American Educational Research Association and American Psychological Association Standards for Educational and Psychological Testing, validity evidence can be broken down into five components: content, response processes, internal structure, relations with other variables, and consequences. 18 Our study takes into account content evidence and assesses relations with other variables evidence. The content evidence comes into play with some of the simulator metrics/variables that we selected, which are either based on published society recommendations 19 (e.g., average withdrawal time) or structured curriculum objectives 20 (e.g., ability to retroflex). Our primary analysis, however, assesses the relations with other variables evidence, using case log numbers as a marker of clinical endoscopy experience and comparing these to metrics recorded by the simulator.

Another limitation of our study is that the ACGME case logging system has been shown to be limited by some degree of inaccuracy secondary to underreporting and inaccurate logging of procedures by trainees. 21 22 23 One potential alternative to determine one's clinical acumen with respect to endoscopy is to use a validated scoring system, such as the Global Assessment of Gastrointestinal Endoscopic Skills (GAGES) tool. 11 Using the GAGES tool would have also allowed us to compare this to our other variables and collect validity evidence in the response processes domain. Yet, another limitation was in regard to the “easy” colonoscopy and the lack of measurement of total time. For a future study, we would consider ending the “easy” colonoscopy without having participants retroflex to record an accurate total and withdrawal time and have them retroflex in isolation by solely performing the retroflexion maneuver at the start of a “second run” of the “easy” case. A few other limitations were regarding the testing of endoscopy skills. Targeting, the fifth skill tested on the FES examination, was initially excluded as there were no appropriate modules to assess this skill available at the time of study inception. As of November 2018, there are two new modules that have been provided by Simbionix that specifically assess this skill. The last limitation is, unlike the FES examination, which assesses five endoscopic skills in isolation of one another, our two cases required the utilization of multiple skills concurrently. While this may be a more realistic reflection of one's clinical competency in regard to actual endoscopy, poor performance with one skill may have affected their performance in another area. We limited this effect by eliminating outcome measures which could be affected by multiple skills.

A future step would be to further analyze virtual colonoscopy performance within a resident cohort for any potential significant differences with regard to targeting ability—the fifth FES skill that can now be assessed on the simulator.

## Conclusion

In conclusion, we did not find correlations between resident endoscopy experience and the main metrics of colonoscopy—completion of colonoscopy, the ability to successfully retroflex, and withdrawal time. There were also limited correlations found with resident endoscopy experience and continuous metrics. As the FES examination is used for general surgery residency programs throughout the country, it is imperative to understand the real utility of simulators for surgical training evaluation.
